# Ferroptosis in myocardial infarction: not a marker but a maker

**DOI:** 10.1098/rsob.200367

**Published:** 2021-04-21

**Authors:** Xiao-dong Wang, Sheng Kang

**Affiliations:** Department of Cardiology, Shanghai East Hospital, Tongji University, Jimo Road 150, Shanghai 200120, People's Republic of China

**Keywords:** iron, ferroptosis, myocardial infarction, cellular metabolism, lipid peroxidation

## Abstract

Identification of effective cardiac biomarkers and therapeutic targets for myocardial infarction (MI) will play an important role in early diagnosis and improving prognosis. Ferroptosis, a cell death process driven by cellular metabolism and iron-dependent lipid peroxidation, has been implicated in diseases such as ischaemic organ damage, cancer and neurological diseases. Its modulators were involved in transferrin receptor, iron chelator, clock protein ARNTL, etc. Its mechanisms included the inhibition of system X_C_^−^, diminished GPX4 activity, change of mitochondrial voltage-dependent anion channels and rising intracellular reactive oxygen species level. Further, the inhibitors of apoptosis, pyroptosis and autophagy did not prevent the occurrence of ferroptosis, but iron chelating agents and antioxidants could inhibit it. Noticeably, ferroptosis is an important pattern of cardiomyocyte death in the infarcted area, which may play a vital role in support of the myocardial pathological process of heart disease. However, the molecular mechanism of ferroptosis in the pathogenesis and the development of MI is not clear. Therefore, a greater depth of exploration of the mechanism of ferroptosis and its inhibitors will undoubtedly improve the pathological process of MI, which may be expected to identify ferroptosis as novel diagnostic and therapeutic targets of MI.

## Graphical abstract

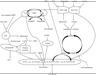

## 1. Introduction

Myocardial infarction (MI) is a pathological process in which the local myocardium is necrotic due to severe persistent ischaemia and hypoxia after coronary occlusion. MI is the primary cause of sudden cardiac death in the world, and the local ischaemia induces extensive tissue injury, heart failure and other complications [1]. Identification of effective cardiac biomarkers and therapeutic targets for MI will play an important role in early diagnosis and the improving prognosis.

Iron is not only the fourth richest element in Earth's crust, but also vital to cell survival since it is part of the Fe–S cluster proteins or as part of the Heme molecule of haemoglobin and myoglobin. In addition, the free divalent iron greatly accelerates lipid peroxidation of saturated fatty acids through Fenton chemistry reaction in humans, and iron also participates in the process of oxidative phosphorylation (OXPHOS) of mitochondria and its productions of reactive oxygen species (ROS) and adenosine triphosphate (ATP). ATP production via OXPHOS is instead needed for cell function; however, lipid peroxidation and ROS will kill the cells. When the oxidation caused by iron deposition exceeds the antioxidant capacity of cells, it is attributed to produce oxidative stress in cells, which directly and/or indirectly damages macromolecular proteins, nucleic acids and lipids, and triggers cell injury and death. The new pattern of cell death is termed ferroptosis [2].

**Figure 1.**
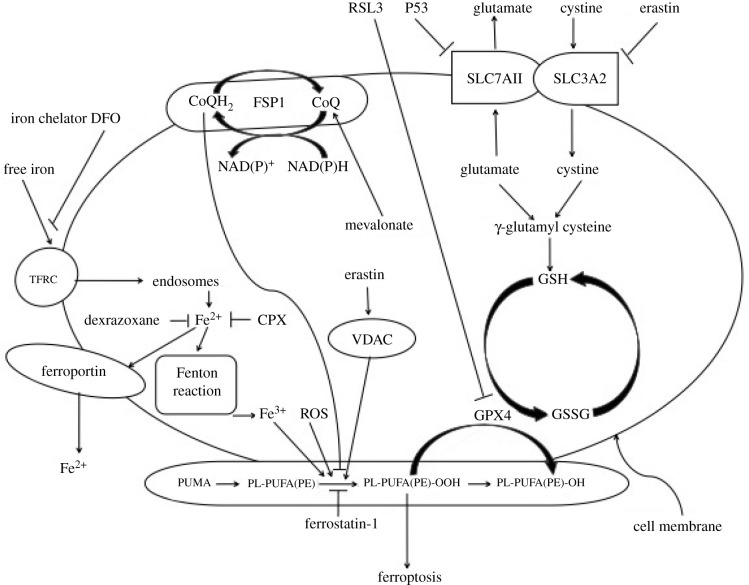
The sketch of ferroptosis mechanism. SLC7A11, together with SLC3A2, encodes the heterodimeric amino acid transport System Xc^−^, a cystine/glutamine antiporter, and deprives cells of glutathione. Glutathione is a necessary substrate for GPX4, which uses glutathione to eliminate lipid peroxides formed in phospholipids containing PUFAs and prevents cell death. Ferroptosis is a newly identified pattern of cell death mediated by iron-induced lipid perxocidation in concomitance with the decreased activity/amount of GPX4. On the other hand, transferrin and transferrin receptor, which import iron from the extracellular environment, are required for ferroptosis. Free divalent iron (Fe^2+^) is turned into Fe^3+^ by Fenton chemistry reaction in cytoplasm, which propagates the peroxidation reaction. Notably, the myristoylation recruits FSP1 to the plasma membrane where it functions as an oxidoreductase that reduces CoQ 10, which acts as a lipophilic radical-trapping antioxidant that halts the propagation of lipid peroxides, thus FSP1 acts as a key component of a nonmitochondrial CoQ antioxidant system. Additionally, Fer-1 is a lipid antioxidant that inhibits ferroptosis. DFO binds Fe^2+^ mainly extracellularly, while dexrazoxane and CPX bind Fe^2+^ intracellularly. Ferroptosis inducers such as Ras Selective Lethal 3 (RSL3), directly inhibit GPX4, thus triggering the accumulation of lipid ROS and resulting in cell death.

It is necessary for ferroptosis to keep the concomitant effects such as drug treatments, inhibition of glutathione peroxidase 4 (GPX4) activity and other concomitant effects. In its modulators, transferrin receptor 1 (TFRC) is the coding gene of transferrin receptor; cellular uptake of free iron (Fe^3+^) occurs via receptor-mediated endocytosis of ligand-occupied transferrin receptor (formation of transferrin-iron complex) into specialized endosomes. Fe^3+^ is converted to Fe^2+^ in endosomes by the metalloreductase six-transmembrane epithelial antigen of prostate 3 (Steap3) and released from endosomes by divalent metal transporter 1 (DMT1) [3,4] but it may promote ferroptosis by upregulating TFRC. Again, free divalent iron (Fe^2+^) is turned into Fe^3+^ by Fenton chemistry reaction in the cytoplasm, which propagates the peroxidation reaction; therefore it is the Fenton reaction, not Fe^3+^ that actives lipid peroxidation. Because the most chemically reactive species of activated oxygen is hydroxyl radical (OH^−^), which is a highly mobile, water-soluble form of ROS that can initiate lipid peroxidation. Fenton and Fenton-like reactions are the main sources of hydroxyl radical formation [5]. Subsequently, the generated Fe^3+^ and the increasing ROS activate lipoxygenases that damage cellular membranes, especially for phosphatidylethanolamine-containing polyunsaturated fatty acids (PUFAs) [4–6] (figure 1). On the other hand, transferrin and transferrin receptor, which import iron from the extracellular environment, are required for ferroptosis. A recent study shows that iron can still accumulate in the liver without transferrin and cause ferroptosis, and slc39a14 functions as the hepatic transport of iron [7]. Lately, ferritinophagy is an autophagic phenomenon that specifically involves ferritin to release intracellular free iron. A recent report demonstrates that ferritinophagy is required for the induction of ferroptosis by the degradation of ferritin [8]. Additionally, the loss of cardiac Ferritin H facilitates cardiomyopathy via slc7a11-mediated ferroptosis [9].

Noticeably, the lipophilic iron chelator directly inactivates the iron-containing enzyme that promotes membrane lipid oxidation, permeates cell membrane and chelates the free ‘redox' iron pool, which reduces the products of catalytic soluble and lipid-free radicals in the iron pool to prevent cell ferroptosis. Alternatively, deferoxamine (DFO), the membrane-impermeable iron chelator, also prevents ferroptosis by chelating free iron (figure 1). Hence, the iron chelator prevents iron from transferring electrons to oxides and inhibits the generation of reactive oxygen species, thereby restricting ferroptosis [10]. Lately, ‘clockophagy', the selective degradation of the core circadian clock protein ARNTL (Aryl Hydrocarbon Receptor Nuclear Translocator Like Protein) by autophagy, facilitates ferroptosis induction [11].

Ferroptosis is generally caused by iron-dependent lipid peroxidation [12]. Regarded as programmed cell death, ferroptosis is involved in lipid metabolism, amino acid metabolism and iron metabolism. It is different from other patterns of cell death in biochemistry and morphology. In its biochemistry, ferroptosis is emerged with glutathione depletion, decreased cystine intake, iron deposition, rising lethal-free radicals and lipid peroxides [13]. On the other hand, ferroptosis sensitivity is also modulated by several other pathways and processes, including the mevalonate pathway. Since this pathway leads to the production of coenzyme Q10, its rate-limiting enzyme is HMG CoA reductase, which is crucial in myocardial infarction.

In its mechanisms, the inhibition of system X_C_^−^ (membrane Na^+^-dependent cysteine-glutamate exchange transporter) can block the transport of intracellular glutamate to extracellular and extracellular cystine to intracellular, which induces ferroptosis. However, some cells make use of the transsulfuration pathway to biosynthesize cysteine from methionine and therefore bypass the requirement for cystine import via the cystine/glutamate antiporter system X_C_^−^, so this pathway is required for cellular metabolism. On the other hand, when erastin, the inducer of ferroptosis, depletes intracellular glutathione, which reduces the GPX4 activity and increases the iron-dependent ROS level in the cytoplasm and lipids, subsequently starting ferroptosis. Therefore, the degradation of glutamate via glutaminolysis is also a fuel of ferroptosis; glutaminolysis-targeted therapy may be effective in treating organ damage mediated by ferroptosis. The inhibition of glutaminolysis has been shown to attenuate ischaemia/reperfusion-induced heart damage [14].

Noticeably, erastin also binds to mitochondrial voltage-dependent anion channels (VDAC2 and VDAC3), changes the membrane permeability, slows down the oxidation of reduced form of nicotinamide-adenine dinucleotid (NADH), changes the iron selectivity of VDAC, whose channel only admits the cations to enter mitochondria, and causes mitochondrial dysfunction and oxidant release, and contributes to oxidation-dependent non-apoptotic cell death (i.e. ferroptosis [15]). In addition, the tumour suppressor gene (p53) increases the intracellular ROS level and triggers the stress, which eventually induces ferroptosis [16] (figure 1).

In the cellular morphology of ferroptosis, the cell membrane appeared rupture and vesicle; the mitochondria size was decreased; the double layers of mitochondria became thicker and denser; the mitochondrial ridges were decreased and even disappeared. The shape of the nucleus was natural without inside chromatin agglutination. The inhibitors of apoptosis, pyroptosis and autophagy did not prevent the occurrence of ferroptosis, but iron chelating agents and antioxidants could inhibit it [2].

Ferroptosis occurs not only in cancer cells [2], but also in neurons [17], even in cardiomyocytes, for example, with development of the reperfusion injury caused by revascularization after coronary occlusion, the cardiomyocytes arise ferroptosis and release inflammatory mediators to aggravate the heart injury. Notably, it has been found that doxorubicin can induce haemoglobin degradation and free iron release in the heart, and provoke ferroptosis in cardiomyocytes, which triggers heart failure [18]. Further, several clinical studies have shown that myocardial iron is an important independent risk factor for left ventricular remodelling after MI [19,20].

Apparently, searching new molecular targets for ferroptosis is gradually becoming the focus in the field of cardiovascular research. Importantly, quantitative proteomic analysis shows that the downregulation of GPX4 in MI contributes to the ferroptosis of cardiomyocytes [21]. However, Puerarin appears to alleviate ferroptosis and prevent heart failure via inducing ferritin production, reducing ROS and NOX4 (Non-phagocytic cell oxidase 4) [22]. In addition, Ferrostatin-1 (Fer-1), a ferroptosis inhibitor, reduces cardiomyocyte death, prevents the recruitment of neutrophils after heart transplantation, decreases the infarct size, diminishes left ventricular remodelling and improves left ventricular systolic function [23]. Thus, ferroptosis is an important pattern of cardiomyocyte death in the infarcted area, which may play a vital role in support of the myocardial pathological process of heart disease [4].

Ferroptosis, a cell death process driven by cellular metabolism and iron-dependent lipid peroxidation, has been implicated in diseases such as ischaemic organ damage; however, the molecular mechanism of ferroptosis in the pathogenesis and development of MI is not clear. Therefore, a greater depth of exploration of the mechanism of ferroptosis and its inhibitors will undoubtedly improve the pathological process of MI, which may be expected to identify ferroptosis as novel diagnostic and therapeutic targets of MI.

## Abbreviations

ARNTL, aryl hydrocarbon receptor nuclear translocator like protein; ATP, adenosine triphosphate; CoQ, coenzyme Q; CPX, ciclopirox; DFO, deferoxamine; DMT1, divalent metal transporter 1; Fer-1, ferroptosis inhibitor Ferrostatin-1; FSP1, ferroptosis-suppressor-protein 1; GPX4, glutathione peroxidase 4; MI, myocardial infarction; NADH, reduced form of nicotinamide-adenine dinucleotid; NOX4, Non-phagocytic cell oxidase 4; OXPHOS, oxidative phosphorylation; p53, tumour suppressor gene; PUFA, polyunsaturated fatty acid; ROS, reactive oxygen species; RSL3, Ras synthetic lethal 3; Steap3, metalloreductase six-transmembrane epithelial antigen of prostate 3; System X_C_^−^, membrane Na^+^-dependent cysteine-glutamate exchange transporter; TFRC, transferrin receptor 1; VDAC, voltage-dependent anion channels.

## Data accessibility

This article has no additional data.
